# Isolation of *Streptomyces* spp. Exhibiting Potent Antibiofilm Activity Against Clinically Isolated Bacterial Strains

**DOI:** 10.1155/ijm/4796619

**Published:** 2025-05-29

**Authors:** Kochar I. Mahmood, Hastyar H. Najmuldeen, Kameran M. Ali, Laila I. Faqe Salih, Ayad M. Ali, Shwan K. Rachid

**Affiliations:** ^1^Medical Laboratory Science Department, Charmo University, Chamchamal, Iraq; ^2^Medical Laboratory Analysis Department, Cihan University Sulaimaniya, Sulaymaniyah, Iraq; ^3^Department of Biology, University of Sulaimani, Sulaymaniyah, Iraq; ^4^Medical Lab Technology Department, Garmian Polytechnic University, Kalar, Iraq; ^5^Department of Chemistry, College of Science, University of Garmian, Kalar, Iraq; ^6^Kurdistan Institution for Strategic Studies and Scientific Researches, Sulaymaniyah, Iraq

**Keywords:** antibiofilm activity, biofilm inhibition, multidrug resistance, secondary metabolites, *Streptomyces albogriseolus*

## Abstract

The increasing threat of antimicrobial resistance (AMR) highlights the urgent need for alternative therapeutic strategies, particularly those targeting microbial virulence factors like biofilm formation. This study aimed to isolate and identify *Streptomyces* species with potential antibiofilm activity against clinically relevant biofilm-producing bacterial pathogens. *Actinomycetes* were isolated from soil samples, cultured on Gause's synthetic agar (GSA) and identified through 16S rRNA gene sequencing. Clinically isolated pathogenic bacteria, including *Proteus mirabilis, Escherichia coli, Klebsiella oxytoca, Acinetobacter baumannii,* and *Klebsiella pneumoniae*, were identified using the VITEK 2 system. The antibiofilm and antibacterial activities of the bioactive compounds extracted from *Streptomyces* spp. were assessed using the agar plug diffusion method and quantitative biofilm assays with crystal violet staining. Among the isolated *Streptomyces* strains, *Streptomyces albogriseolus* was identified as a promising producer of bioactive metabolites. The isolate exhibited 99% similarity to strain NBRC 3709 based on 16S rRNA gene sequencing. The crude extract at a concentration of 20 mg/mL demonstrated significant antibacterial activity, with inhibition zones of 11.9 mm against *K. pneumoniae* and 15.1 mm against *E. coli*. Moreover, the extract significantly reduced biofilm formation in *A. baumannii* and *E. coli*. A lower antibiofilm effect was also observed against *K. pneumoniae, P. mirabilis,* and *K. oxytoca*, with *K. oxytoca* exhibiting the weakest biofilm inhibition. In conclusion, secondary metabolites from *S. albogriseolus* display significant antibiofilm activity against drug-resistant pathogens, with efficacy varying by bacterial species and extract concentration. These findings underscore the potential of *Streptomyces*-derived metabolites as promising candidates for combating biofilm-associated infections. Further studies are recommended to explore their mechanism of action and optimize their potential therapeutic application.

## 1. Introduction

The rapid rise of antimicrobial resistance (AMR) has emerged as one of the most critical public health crises globally [1]. The persistence of AMR is exacerbated by its significant burden on healthcare systems, leading to increased treatment costs, limited therapeutic options, and elevated rates of morbidity and mortality due to bacterial infections [1–4]. Despite concerted efforts over recent decades to mitigate AMR, the global trends indicate no slowdown in its progression [2]. The World Health Organization (WHO) recognizes AMR as one of the Top 10 global health threats to humanity [5].

Biofilm formation is a major driver of AMR, as biofilm-associated infections exhibit heightened tolerance to antibiotics, immune clearance, and disinfectants [6, 7]. These infections often require antibiotic concentrations up to 1000 times higher than those effective against planktonic bacteria, making treatment challenging [8, 9]. Despite new therapeutic agents, many have undesirable side effects, while drug-resistant pathogens continue to outpace antimicrobial development [10–13]. This growing threat necessitates alternative strategies that target biofilm formation itself rather than relying solely on conventional antimicrobials [1].

Microbial natural products, especially from *Streptomyces*, are a key source of diverse antimicrobial agents. Over the past century, they have played a crucial role in antibiotic discovery and drug development [14–17]. In addition to antibiotics, they produce compounds with antibiofilm, antifungal, antiviral, anticancer, and antioxidant activities. Notably, *Streptomyces* account for nearly 80% of clinically used antibiotics [18–22]. However, many of their bioactive compounds remain unexplored, underscoring the urgency to discover new antimicrobial agents, particularly those with antibiofilm properties [15]. Despite extensive studies on *Streptomyces* species, *S. albogriseolus* remains relatively unexplored for its bioactive potential. This study aims to evaluate the antibacterial and antibiofilm potential of secondary metabolites from *S. albogriseolus* against multidrug-resistant pathogens. The objectives include the isolation and molecular identification of *S. albogriseolus*, preparation of crude extracts, and *in vitro* assessment of antibacterial and antibiofilm activities.

## 2. Methodology

### 2.1. Soil Sampling

A total of 23 soil samples were collected from Koya and Sulaymaniyah, Kurdistan Region of Iraq, encompassing agricultural, mountainous, and natural habitats in a semiarid climate. To optimize Actinobacteria isolation, samples were taken from a 20–40-cm depth after removing the top 3 cm, following Pandey et al. [23].

### 2.2. Isolation of Actinomycetes

Soil samples (1 g) were suspended in 9 mL of distilled water and heated at 55°C for 6 min to suppress non-spore-forming bacteria [24]. After cooling, suspensions were serially diluted up to 10^−5^. Actinomycetes were isolated on Gause's synthetic agar (GSA) supplemented with 50 mg/mL nystatin and 25 mg/mL nalidixic acid to inhibit fungal and Gram-negative bacterial contamination [25]. Dilutions (0.1 mL) were spread on GSA plates and incubated at 28°C for 7 days. Colonies were subcultured on fresh GSA for an additional 7 days to obtain pure isolates [26]. Spores from pure cultures were harvested, washed, and stored in sterile 20% glycerol at −80°C [27].

#### 2.2.1. Primary Screening of Antibacterial Activity (Agar Plug Diffusion Method)

The antibacterial activity of isolated Actinomycetes was screened using the agar plug diffusion method. Pure cultures were grown on GSA at 28°C for 7 days to allow metabolite diffusion. Agar plugs (8 mm) were aseptically removed and placed on Mueller-Hinton agar preinoculated with clinically isolated pathogens, including *Proteus mirabilis, Escherichia coli, Klebsiella oxytoca, Acinetobacter baumannii*, and *Klebsiella pneumoniae*. Plates were incubated at 37°C for 24 h, and inhibition zones (mm) were measured. The most potent isolate, based on the largest inhibition zone, was selected for antibiofilm screening. This method was adapted from [28, 29].

### 2.3. Molecular Identification of *Streptomyces*

#### 2.3.1. Genomic DNA Isolation

The most potent antimicrobial-producing *Streptomyces* spp. was selected and cultured aerobically in Tryptic Soy Broth (TSB) at 28°C, 200 rpm for 48 h. Genomic DNA was extracted using the Presto Mini gDNA Bacteria Kit (Geneaid Biotech Ltd.), and its concentration and purity were assessed with a NanoDrop spectrophotometer (Thermo Fisher Scientific, United States).

#### 2.3.2. Polymerase Chain Reaction (PCR) Amplification of *Streptomyces* 16S rRNA

The 16S rRNA gene was amplified using specific primers: forward 5⁣′-GACAAGCCCTGGAAACGGGGT-3⁣′ and reverse 5⁣′-GCTCGTGTCGTGAGATGTTGGG-3⁣′ (Macrogen, South Korea) [9]. The 30-*μ*L PCR reaction included 10-*μ*L Ready-to-use EasyTaq PCR SuperMix (2×), 1.5 *μ*L of each primer (10 pmol/*μ*L), 3-*μ*L genomic DNA, and 14-*μ*L ddH₂O. The thermocycler conditions were as follows: initial denaturation at 95°C for 5 min, followed by 35 cycles of denaturation (95°C, 30 s), annealing (60°C, 40 s), extension (72°C, 45 s), and a final extension at 72°C for 3 min.

#### 2.3.3. Gel Electrophoresis and Sequencing

PCR products (~900 bp) were resolved on a 1.5% agarose gel in 1x TBE buffer at 90 V for 1 h, visualized under UV light, and sequenced using the Sanger method (Daejeon, South Korea). Sequences were analyzed with BioEdit v7.2.5 and compared to GenBank using BLAST. Identified 16S rRNA sequences were submitted to GenBank for accession numbers.

#### 2.3.4. Phylogenetic Analysis

The phylogenetic analysis of *Streptomyces albogriseolus* strain 8K (ON364557.1) was conducted using MEGA 11 to determine its evolutionary relationship with other *Streptomyces* strains retrieved from NCBI GenBank. The 16S rRNA gene sequence of strain 8K was aligned with reference sequences using ClustalW with default parameters.

A phylogenetic tree was constructed using the neighbor-joining (NJ) method, and evolutionary distances were computed using the maximum composite likelihood model. To ensure the robustness of the inferred relationships, 1000 bootstrap replications were performed, and bootstrap values above 50% were displayed at respective nodes. The final tree was visualized and formatted in MEGA 11.

### 2.4. Isolation of Pathogenic Bacteria

Pathogenic bacteria were collected from clinical samples (urine, wounds, blood, burns, catheters, and respiratory aspirates) and hospital environments (burn units, wards, operating rooms, and bathrooms) at Shar Hospital, Sulaimani, between December 2022 and May 2023.

#### 2.4.1. Identification by VITEK 2 System

Clinical isolates were identified using VITEK 2 ID-Gram-negative bacilli cards (BioMérieux, France) per the manufacturer's protocol.

### 2.5. Culture Fermentation and Extraction

Fermentation was initiated by inoculating 100 mL of TSB with 100 *μ*L of spore stock (~10^8^ spores/mL) in 500-mL Erlenmeyer flasks [30], followed by incubation at 28°C, 200 rpm for 48 h. The culture was adjusted to OD₆₀₀ = 0.1, and 5% (*v*/*v*) of the inoculum was transferred to two 5-L fermenters (4.5-L working volume) for a 10-day incubation.

Metabolites were extracted twice with ethyl acetate (1:1 *v*/*v*), and the organic layers were concentrated under reduced pressure using a rotary evaporator (Heidolph, GmbH & Co., KG, Germany) [31]. The final crude extract (1.2 g, reddish-brown) was dissolved in methanol (20 mg/mL) and stored at −20°C.

### 2.6. Quantitative Biofilm Formation Assay

Biofilm formation was assessed using the microtiter plate method described by Babapour et al. [32]. Five pathogenic bacterial isolates in triplicates were tested, each as an independent biological replicate. Bacterial cultures were grown in nutrient broth to OD₆₀₀ = 0.1 and then diluted 1:100 in fresh medium. Diluted cultures (200 *μ*L) were added to 96-well plates and incubated at 37°C for 24 h. Each experiment included three biological replicates per pathogen, with three technical replicates per condition.

After incubation, wells were washed with phosphate-buffered saline (PBS), air-dried, and stained with 0.1% crystal violet for 15 min. Excess stain was removed, and biofilm-bound dye was solubilized using ethanol–acetone (80:20 *v*/*v*). Absorbance was measured at 595 nm using a microplate reader.

Biofilm formation was classified based on OD₅₉₅ values relative to controls [33, 34]:

• **Weak**: ODT ≤ 2ODC
• **Moderate**: 2ODC < ODT ≤ 4ODC
• **Strong**: ODT > 4ODC

where ODC is the optical density of the negative control and ODT represents the test samples.

### 2.7. Antibiofilm Screening Using Crystal Violet Staining

The antibiofilm activity of the crude extract was evaluated in 96-well plates following previous protocols [35–37]. Five different pathogenic bacterial isolates (*P. mirabilis*, *E. coli*, *K. oxytoca*, *A. baumannii*, and *K. pneumoniae*) were used. Each experiment included three biological replicates per pathogen, with three technical replicates per condition to ensure reproducibility. The crude extract (20 mg/mL) and methanol (negative control) were added to fresh nutrient broth containing bacterial cultures at a standardized initial cell density of OD_600_ = 0.1 before being diluted 1:100. Ciprofloxacin (10 *μ*g/mL) was used as a positive control, as it has been reported to exhibit antibiofilm activity. Biofilm formation was assessed after 24 h of incubation at 37°C. Wells were washed with PBS to remove planktonic cells, and biofilm cells were stained with 0.1% crystal violet. The biofilm mass was solubilized with ethanol-acetone (80:20 *v*/*v*) and measured at 595 nm using a microplate reader.

To quantify biofilm inhibition, OD_595_ values were measured before and after treatment, with reductions expressed as absolute values and logarithmic reductions to provide a standardized comparison. The logarithmic reduction was calculated as follows:

 $$\begin{array}{c} \text{Logarithmic Reduction}=\log_{10}\left(\text{OD Control}-\text{OD Treated}\right) \end{array}$$

OD Control represents the optical density of the biofilm in the untreated group, while OD Treated refers to the optical density of the biofilm in the treated group (exposed to the crude extract). This method enables a precise comparison of biofilm inhibition across different strains. The results demonstrate a significant reduction in biofilm formation without affecting bacterial growth.

### 2.8. Statistical Analysis

GraphPad Prism software Version 6 (GraphPad, California, United States) was used for statistical analysis. The experimental results were expressed as the mean ± standard deviation (SD) from three independent biological replicates. Group comparisons were performed using one-way analysis of variance (ANOVA) followed by Dunnett's multiple comparisons test to assess the significance of differences between treated and control groups. A *p* value of less than 0.05 was considered statistically significant. Statistical significance was denoted as follows: a single star (⁣^∗^) for *p* < 0.05, two stars (⁣^∗∗^) for *p* < 0.01, three stars (⁣^∗∗∗^) for *p* < 0.001, and four stars (⁣^∗∗∗∗^) for *p* < 0.0001.

## 3. Results

### 3.1. Agar Plug Diffusion

The antibacterial activity of 12 Actinomycete isolates, including *S. albogriseolus* (Isolate-5), was evaluated against five clinically relevant pathogens: *P. mirabilis, A. baumannii, E. coli, K. oxytoca*, and *K. pneumoniae*. The isolates exhibited varying inhibition zones against the tested pathogens. Notably, Isolate-5 demonstrated the strongest antibacterial activity, with inhibition zones of 16 ± 2.7 mm against *A. baumannii*, 15 ± 2.49 mm against *K. oxytoca*, 12 ± 2.9 mm against *E. coli*, 11 ± 2.2 mm against *P. mirabilis*, and 10 ± 1.1 mm against *K. pneumoniae* (Table 1).

Among the remaining isolates, Isolates-8 and Isolates-2 exhibited notable activity, particularly against *P. mirabilis* and *A. baumannii*, whereas Isolate-11 displayed the weakest inhibition (Table 1). The positive control, ciprofloxacin (5 *μ*g/disc), produced inhibition zones ranging from 13 ± 1.72 to 19 ± 4.26 mm, confirming the overall efficacy of the isolates. These findings suggest that *S. albogriseolus* and other Actinomycetes could serve as potential sources for novel antibacterial agents, particularly against multidrug-resistant pathogens.

### 3.2. Molecular Identification

**Figure 1:**
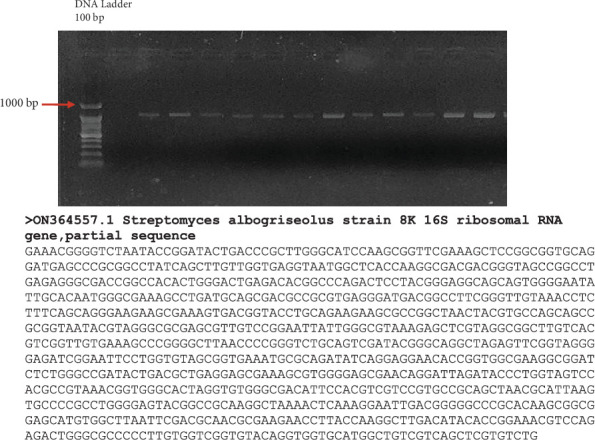
Agarose gel electrophoresis of the PCR-amplified 16S rDNA fragment, visualized under UV light, alongside the FASTA-formatted nucleotide sequence of the amplified *Streptomyces albogriseolus* 16S rDNA.

To confirm its identity, PCR was performed, successfully amplifying a 902-base pair (bp) fragment of the 16S ribosomal DNA (rDNA). Sequence analysis of the amplified region demonstrated 99% similarity to *S. albogriseolus* strain NBRC 3709, confirming a close genetic relationship with the reference strain. The amplified sequence has been deposited in GenBank (NCBI) under accession number ON364557 (Figure 1).

**Figure 2:**
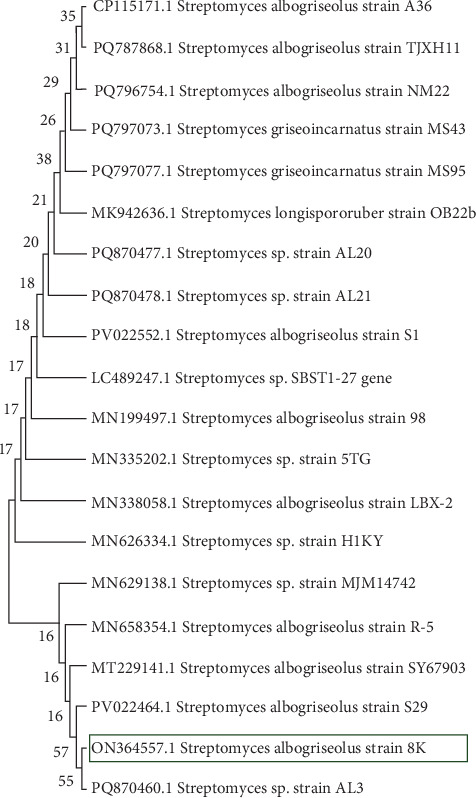
Phylogenetic tree analysis of *Streptomyces albogriseolus* isolates based on 16S rRNA gene sequences using MEGA 11.0.

The bootstrap-supported phylogenetic tree (Figure 2) illustrates the evolutionary placement of *S. albogriseolus* strain 8K (ON364557.1) in relation to other closely related strains. The analysis revealed that strain 8K is closely related to *Streptomyces* sp. strain AL3 (PQ870460.1), forming a distinct cluster.

Further, strain 8K and strain AL3 belong to a larger *S. albogriseolus* subgroup, including *S. albogriseolus* strain S29 (PV022464.1), *S. albogriseolus* strain SY67903 (MT229141.1), and *S. albogriseolus* strain R-5 (MN658354.1). This clustering suggests that strain 8K shares a recent common ancestor with these *S. albogriseolus* strains, supporting its classification within the *S. albogriseolus* clade. The bootstrap values within the clade indicate the statistical support for these evolutionary relationships, reinforcing the phylogenetic closeness between strain 8K and strain AL3.

### 3.3. Biofilm Formation and Antibiofilm Activity

The tested bacterial isolates exhibited varying degrees of biofilm formation. *K. pneumoniae* displayed the highest biofilm production (OD = 4.428 ± 0.193), followed by *P. mirabilis* (OD = 4.063 ± 0.1) and *E. coli* (OD = 3.21 ± 0.073). *A. baumannii* demonstrated moderate biofilm adherence (OD = 3.086 ± 0.19), whereas *K. oxytoca* exhibited the lowest biofilm intensity (OD = 1.79 ± 0.162) among the tested isolates (Table 2).

### 3.4. Antibiofilm Activity

**Figure 3:**
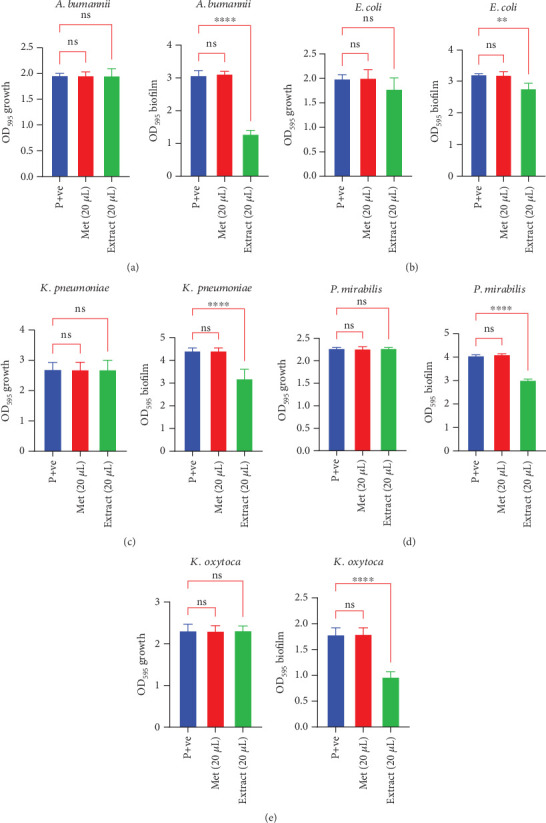
Biofilm inhibition in tested pathogens by the crude extract from *Streptomyces albogriseolus* compared to the positive control. The figure illustrates the reduction of biofilm produced by pathogenic bacteria, including (a) *A. baumannii*, (b) *E. coli*, (c) *K. pneumoniae*, (d) *P. mirabilis*, and (e) *K. oxytoca*, using 20 *μ*L of the crude extract from fermented *S. albogriseolus*. The impact of both methanol (Met.) and the extract on bacterial growth is shown, alongside their influence on biofilm formation. Statistical significance is indicated by stars, with “ns” denoting no significant effect (p + ve: OD_595_ of biofilm).

The secondary metabolite extracted from *S. albogriseolus* exhibited a strong antibiofilm effect against the tested pathogens, significantly reducing biofilm formation without impairing bacterial growth. When 20 *μ*L of the crude extract (20 mg/mL) was applied, *A. baumannii* biofilm formation was markedly reduced (OD = 1.294 ± 0.15, *p* < 0.05) compared to the untreated control (OD = 3.086 ± 0.19), while bacterial growth remained unchanged (OD = 1.96 ± 0.166 vs. 1.96 ± 0.086, *p* > 0.05) (Figure 3a). Similarly, a significant reduction in biofilm formation was observed in *E. coli* (OD = 2.764 ± 0.219 vs. control OD = 3.21 ± 0.07, *p* < 0.05), with no substantial effect on bacterial growth (OD = 1.78 ± 0.26) (Figure 3b). Comparable inhibitory effects were recorded for *K. pneumoniae, P. mirabilis,* and *K. oxytoca* (Figures 3c, 3d, and 3e). These findings highlight the extract's potential as an antibiofilm agent, effectively disrupting biofilm formation while preserving bacterial viability.

### 3.5. Minimum Inhibitory Concentration (MIC) Analysis

Following the confirmation of antibiofilm activity, the MIC was determined. MIC was assessed by testing various volumes of the crude extract (5–30 *μ*L) from a 20-mg/mL stock solution, in triplicate. The applied volumes corresponded to final extract concentrations ranging from 500 (5 *μ*L) to 3000 *μ*g/mL (30 *μ*L) in a total well volume of 200 *μ*L.

**Figure 4:**
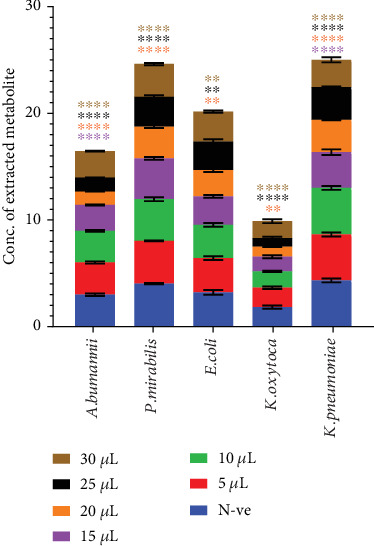
Minimum inhibitory concentration (MIC) of the secondary metabolite against biofilm formation in tested pathogens. The figure illustrates the effect of varying metabolite concentrations (500–3000 *μ*g/mL) on biofilm formation in *A. baumannii, P. mirabilis, E. coli, K. oxytoca*, and *K. pneumoniae*. Data are presented as mean OD_595_ values, with statistically significant differences (*p* < 0.0001) indicated by stars.

Figure 4 presents the MIC for biofilm inhibition, with different extract volumes represented by distinct colors. Statistically significant effects (*p* < 0.0001) are indicated by stars on the graph columns. These results highlight the concentration-dependent nature of the secondary metabolite's antibiofilm activity, underscoring its potential for further dose optimization studies.

## 4. Discussion

The antimicrobial activity observed in our study aligns with previous research on bioactive *Streptomyces* species. The inhibition zones produced by *S. albogriseolus* ranged from 10 to 16 mm against the tested pathogens, which is within the range reported for other bioactive *Streptomyces* strains. This further supports the potential of Actinomycetes as a rich source of antimicrobial compounds. A previous study by Chang et al. [17] reported that among the 116 Actinomycete-like isolates obtained from a water pipe, one isolate exhibited significant antimicrobial activity against a broad spectrum of microbial test strains. This included Gram-positive bacteria (*Staphylococcus aureus* ATCC 12145), Gram-negative bacteria (*Pseudomonas aeruginosa* ATCC 11633 and *E. coli* DH5*α*), as well as fungal pathogens (*Saccharomyces cerevisiae* ATCC 9763, *Candida albicans* ATCC 10231, and *Aspergillus niger* ATCC 16404). These findings highlight the potential of Actinomycetes as a valuable source of bioactive compounds with broad-spectrum antimicrobial properties.

The primary focus on *S. albogriseolus* is due to its relative novelty in antibiofilm research, as it remains underexplored compared to well-characterized *Streptomyces* species. While the *Streptomyces* genus is renowned for producing diverse bioactive metabolites, emerging evidence suggests that *S. albogriseolus* may harbor antimicrobial and antibiofilm compounds. Additionally, several endophytic *Streptomyces* species have been reported to synthesize antibiotics. Notably, *Streptomyces fulvoviolaceus, Streptomyces caelestis,* and *Streptomyces coelicolor* have demonstrated potent antifungal activity against multiple plant pathogens, including *Phytophthora erythroseptica, Pythium ultimum, Sclerotinia sclerotiorum, Mycosphaerella fijiensis,* and *Rhizoctonia solani* [38]. These findings highlight the diverse bioactive potential of *Streptomyces* species and underscore the importance of further investigating *S. albogriseolus* for its potential antimicrobial applications.

This study provides compelling evidence of the antibiofilm properties of secondary metabolites extracted from *S. albogriseolus*, highlighting their potential as effective agents against biofilm-associated infections. The antibiofilm activity was concentration-dependent, with lower concentrations demonstrating greater efficacy in inhibiting biofilm formation, consistent with previous findings [9]. This underscores the need for dosage optimization, as lower concentrations can disrupt biofilm matrices without inducing bacterial cell death, thereby minimizing the risk of resistance development commonly associated with bactericidal agents.

While our study primarily focused on demonstrating the antibiofilm efficacy of *S. albogriseolus* crude extracts, we recognize the importance of identifying the active compounds. Previous research indicates that *Streptomyces* species commonly produce polyketides, nonribosomal peptides, and alkaloids with antibiofilm properties [39]. Many *Streptomyces*-derived bioactive compounds exhibit antibiofilm activity by interfering with quorum sensing, biofilm matrix production, or bacterial adhesion [35].

The mechanism of action of these metabolites in biofilm inhibition remains to be fully elucidated. While the observed antibiofilm effects are promising, additional studies are necessary to determine whether these metabolites act through novel pathways or mechanisms distinct from previously reported antibiofilm agents. The ability to inhibit biofilm formation without killing the bacteria suggests that these compounds may offer a therapeutic alternative to conventional treatments. However, careful consideration is needed when using these biofilm inhibitors, as high concentrations could stress bacterial cells and potentially trigger adaptive resistance mechanisms. Conversely, higher doses could overwhelm biofilm-forming bacteria, leading to their complete elimination in some cases. Therefore, optimizing the dose for biofilm inhibition, based on the specific pathogen and infection context, is crucial for achieving effective therapeutic outcomes [40].

Further investigation into the concentration-dependent nature of the metabolites' antibiofilm effects revealed differential responses among various pathogens. For example, *A. baumannii* required a minimum volume of 15 *μ*L (1500 *μ*g/mL) for significant biofilm inhibition (*p* < 0.0001), while *P. mirabilis* needed 20 *μ*L (2000 *μ*g/mL) for a similar effect. *E. coli*, however, exhibited a weaker response, with significance achieved only at 20 *μ*L (2000 *μ*g/mL) (*p* < 0.05). *K. oxytoca* demonstrated a substantial reduction in biofilm production at 20 *μ*L (2000 *μ*g/mL) (*p* < 0.0001), while *K. pneumoniae* achieved significant inhibition with just 15 *μ*L (1500 *μ*g/mL) (*p* < 0.0001). These variations underscore the importance of pathogen-specific strategies when designing treatments for biofilm-related infections. The concentration-dependent activity also emphasizes the need for personalized treatment approaches, which could enhance both treatment precision and resource optimization [41, 42].

Several *Streptomyces* species have been documented for their antibiofilm and antimicrobial activities, including *S. coelicolor, S. griseus*, and *S. hygroscopicus*. *S. coelicolor* produces actinorhodin, a redox-active antibiotic that disrupts bacterial cell processes, contributing to biofilm inhibition [43]. *S. griseus* is renowned for producing streptomycin, an aminoglycoside antibiotic that, beyond its primary antibacterial action, has shown efficacy against biofilm-forming bacteria [44]. *S. hygroscopicus* synthesizes rapamycin, an immunosuppressant with indirect effects on microbial biofilms, particularly through modulation of host immune responses [45]. In comparison, our study demonstrates that *S. albogriseolus* exhibits strong antibiofilm activity, significantly reducing biofilm formation in *A. baumannii, E. coli*, and *K. pneumoniae*, with a slightly weaker effect on *K. oxytoca* and *P. mirabilis*. These results suggest that *S. albogriseolus* possesses comparable antibiofilm properties against certain multidrug-resistant pathogens.

Biofilm formation in bacterial pathogens such as *E. coli* and *P. aeruginosa* is regulated by quorum-sensing systems, including *luxS, lasR,* and *rhlR* [46]. Many *Streptomyces* species, including *S. albogriseolus*, produce quorum-sensing inhibitors that disrupt these signaling pathways, thereby interfering with biofilm development [35]. Furthermore, the initial stage of biofilm formation involves bacterial attachment to surfaces, which is mediated by specific adhesion factors. If metabolites produced by *S. albogriseolus* interfere with key adhesion structures such as curli fimbriae in *E. coli* or Type IV pili in *A. baumannii*, this could explain the observed reduction in biofilm formation in our study [47]. These findings suggest that *S. albogriseolus*-derived compounds may have a significant potential as biofilm inhibitors by targeting both quorum-sensing mechanisms and bacterial adhesion processes.

Biofilms are encased in an extracellular polymeric substance (EPS) composed of proteins, polysaccharides, and extracellular DNA, which contribute to their structural integrity and resistance to antimicrobial agents. *Streptomyces*-derived enzymes, such as DNases, proteases, and glycoside hydrolases, play a crucial role in biofilm dispersal by degrading these matrices. In addition to enzymatic degradation, biofilm disruption can be facilitated by antibiofilm peptides and dispersal molecules, including dispersal signals, antimatrix compounds, and sequestration molecules, which collectively weaken biofilm integrity and enhance bacterial susceptibility to treatment [48].

The findings of this study contribute to the development of more focused strategies to combat biofilm-associated infections, highlighting the promising potential of secondary metabolites from *S. albogriseolus* as antibiofilm agents against clinically relevant pathogens. These metabolites significantly reduce biofilm formation without exerting bactericidal effects, positioning them as viable candidates for preventing biofilm-associated infections. Future research should aim to further elucidate the chemical structure of these metabolites and their precise mechanisms of action, which could lead to novel therapeutic strategies for combating biofilm-related infections. Additionally, further studies are needed to characterize the chemical composition of the bioactive compounds and explore their specific mechanisms of biofilm inhibition. Such research will provide critical insights into the clinical potential of these compounds and help refine treatment approaches for biofilm-associated infections.

## Figures and Tables

**Table 1:** Inhibition zone diameters (mm) of secondary metabolites from agar plugs of isolated Actinomycete strains, evaluated using the agar plug diffusion assay against clinically relevant pathogenic bacteria.

**Isolated strains**	**Tested pathogens**				
	***P. mirabilis***	***A. baumannii***	***E. coli***	***K. oxytoca***	***K. pneumoniae***
Isolate-1	6.5 ± 1.3	10.2 ± 2.2	8.0 ± 2.0	9.1 ± 2.0	7.0 ± 1.8
Isolate-2	7.1 ± 1.6	11.0 ± 2.5	8.5 ± 2.3	9.6 ± 2.3	7.5 ± 1.9
Isolate-3	5.8 ± 1.4	9.8 ± 2.3	7.8 ± 2.1	8.8 ± 2.1	6.9 ± 1.7
Isolate-4	6.9 ± 1.7	12.0 ± 2.6	9.2 ± 2.5	10.0 ± 2.4	8.0 ± 2.0
Isolate-5 (*S. albogriseolus*)	11 ± 2.2	16 ± 2.7	12 ± 2.9	15 ± 2.49	10 ± 1.1
Isolate-6	6.2 ± 1.5	10.0 ± 2.4	7.9 ± 2.0	9.5 ± 2.1	7.3 ± 1.8
Isolate-7	6.8 ± 1.6	10.9 ± 2.5	8.4 ± 2.2	10.2 ± 2.4	7.8 ± 1.9
Isolate-8	7.4 ± 1.8	11.3 ± 2.7	8.9 ± 2.3	10.7 ± 2.6	8.5 ± 2.1
Isolate-9	6.9 ± 1.5	10.1 ± 2.4	8.3 ± 2.1	9.9 ± 2.3	7.7 ± 1.9
Isolate-10	6.3 ± 1.4	9.6 ± 2.3	7.8 ± 2.0	8.7 ± 2.2	7.2 ± 1.8
Isolate-11	5.7 ± 1.2	9.0 ± 2.1	7.2 ± 1.9	9.1 ± 2.0	6.8 ± 1.6
Isolate-12	6.4 ± 1.6	9.7 ± 2.3	7.6 ± 2.1	8.5 ± 2.3	7.0 ± 1.7
Positive control (ciprofloxacin)	16 ± 3.6	19 ± 4.26	13 ± 1.72	17 ± 4.5	15 ± 3.7

*Note:* The values for both Isolate-5 and ciprofloxacin are shown in bold to emphasize the notable antibacterial activity of the secondary metabolite produced by the isolate.

**Table 2:** Biofilm formation intensity of clinical bacterial isolates based on optical density measurements (*p* < 0.05).

**Pathogen**	**ODC**	**4**∗** ODC**	**ODT (** **m** **e** **a** **n** **s** ± **SD)**	**Biofilm intensity**
*Acinetobacter baumannii*	0.141	0.564	3.086 ± 0.19	Strong
*Escherichia coli*	0.147	0.588	3.21 ± 0.073	Strong
*Proteus mirabilis*	0.167	0.668	4.063 ± 0.1	Strong
*Klebsiella oxytoca*	0.178	0.712	1.79 ± 0.162	Strong
*Klebsiella pneumoniae*	0.137	0.548	4.428 ± 0.193	Strong

## Data Availability

Data availability is provided if requested.

## Nomenclature

PCR: polymerase chain reaction
rDNA: ribosomal DNA
bp: base pair
OD: optical density
MIC: minimum inhibitory concentration
NCBI: National Center for Biotechnology Information
